# Effect of welfare standards and biosecurity practices on antimicrobial use in beef cattle

**DOI:** 10.1038/s41598-020-77838-w

**Published:** 2020-12-01

**Authors:** Alessia Diana, Valentina Lorenzi, Mauro Penasa, Edoardo Magni, Giovanni L. Alborali, Luigi Bertocchi, Massimo De Marchi

**Affiliations:** 1 Department of Agronomy, Food, Natural resources, Animals and Environment (DAFNAE), University of Padova, Viale dell’Università 16, 35020 Legnaro (PD), Italy; 2 Italian National Reference Centre for Animal Welfare, Istituto Zooprofilattico Sperimentale della Lombardia e dell’Emilia Romagna ‘Bruno Ubertini’ (IZSLER), Via Bianchi 9, 25124 Brescia, Italy

**Keywords:** Antibiotics, Antimicrobial resistance, Animal behaviour

## Abstract

Antimicrobial use (AMU) in livestock species and the associated antimicrobial resistance are a global concern, thus strategies for their reduction and a more judicious use are needed. Previous research has revealed a link between improved animal welfare, biosecurity and AMU reduction in pig and dairy sectors, however, little is known about the beef sector. This study aimed to investigate the impact of welfare standards and biosecurity on AMU in beef cattle. Data on performance traits and AMU were collected over a 3.5 year time from 27 specialised beef farms and a treatment incidence was calculated using the defined daily dose for animals. An on-farm assessment was carried out by assigning a score from 0 (very poor) to 100% (very good) to 3 sections: welfare, biosecurity and emergency management. The highest average score was obtained for the welfare section (76%) followed by emergency management (39%) and biosecurity (24%). This suggests that major focus on strategies for the implementation of biosecurity measures and emergency management is needed, due to the low scores reported. A statistically significant lower AMU was observed with improved level of welfare. These results may be helpful for farm benchmarking and highlight the importance of improved animal welfare for an efficient antimicrobial stewardship.

## Introduction

Animal welfare (welfare) is defined as ‘the physical and mental state of an animal in relation to the conditions in which it lives and dies’^1^, thus emphasising the importance of biological functioning and natural behavioural needs alike for the assessment of animal well-being^2^. The link between animal health and welfare is widely acknowledged^3–5^ as well as the impact that good welfare may have on animal productivity^5,6^. Indeed, reduced stress levels are essential to avoid an impairment of animals’ immune system which in turn would affect their performance and susceptibility to diseases^7^. Similar evidence is also reported for the positive role of improved biosecurity on animal health and welfare. Biosecurity consists of procedures intended to prevent both pathogens entering a farm (external biosecurity) and pathogens spreading within a farm (internal biosecurity)^5,8^. Pandolfi et al.^5^ reported lower prevalence of some disease indicators and negative welfare outcomes, such as severe tail lesions and lameness, in pig farms with high biosecurity and average daily gain (ADG). Ohlson et al.^9^ found an association between lower prevalence of infections with better biosecurity at herd level. In their review, Stokstad et al.^10^ largely discussed about the importance of implementing biosecurity measures to prevent and reduce respiratory diseases in cattle. Hence, both biosecurity and welfare standards are recognized as basic principles of an efficient livestock management^8,11,12^.

Another important aspect of a general farm management is the use of antimicrobials (AMU), since they are useful tools in controlling infectious diseases, especially in intensive farming systems where the spread of pathogens is more likely to occur^13–15^. Despite such an essential role, massive AMU and the associated antimicrobial resistance (AMR) are major concerns worldwide^16,17^. New strategies to advance a more judicious use of medications are needed and providing accurate data on AMU is fundamental to achieve this goal^18^.

Livestock species in EU account for a large proportion of use of antimicrobials^19–21^, making them major contributors to the increase of AMR^16,22,23^. However, differently from dairy, poultry and swine, only few studies explored the potential risk factors of AMU in the beef sector such as farm size, the duration of the fattening period and quarantine of purchased animals^24–26^. In our previous study on Italian beef cattle^27^, we observed that breed was an important source of variation of AMU with Blonde d’Aquitaine and Limousine being at greater risk of treatment than Charolaise. We also reported a wide variability of AMU among farms suggesting, in accordance with other studies^28,29^, the role that distinctive farm-factors like welfare and biosecurity procedures or the farmer-veterinarian relationship^30^ may play on the overall AMU.

The European Innovation Partnership for Agricultural Productivity and Sustainability (EIP-AGRI) emphasised that more research is needed to assess the impact of welfare on AMU^31^. Additionally, there is evidence that high standards of both internal and external biosecurity, and the preventive role played on-farm, may lead to improved animal health and productivity, and in turn to a reduction of AMU^32,33^. For instance, Postma et al.^34^ observed that better biosecurity was linked to a lower AMU in pigs while Raasch et al.^35^ reported a reduction of AMU following the implementation of tailored welfare-friendly procedures on-farm. However, difficulty in applying such changes may be due to farmers’ beliefs surrounding use of medications. For instance, the common perception that antimicrobials are essential to preserve animal health and welfare^36,37^ or farmers’ opinion that the amount of medications used is low and provided only when necessary^38,39^. As a result, farmers may become reluctant in removing antimicrobials and/or changing certain management procedures. However, some studies confuted this belief reporting how even with a high AMU as part of the common farm management, yet pigs were showing poor welfare conditions and health issues^40,41^. Despite the well-known association between biosecurity, welfare standards and animal health and performance^5,10^, only few studies explored their influence on AMU in beef cattle^15,24,42^. For instance, Becker et al.^42^ reported a drastic reduction of AMU in veal calves following an improvement of those management practices considered as responsible of high AMU (e.g. crowding and suboptimal barn climate conditions).

Every year, 70% of beef cattle produced in Italy are fattened in specialised fattening farms in Veneto region, in the north-east of the country. Beef producers purchase calves from other EU countries, mostly from France which is the principal supplier and accounts for c. 90% of imported animals^43^. The main purchased breeds are Charolaise and Limousine, while only a minority of the animals belongs to other breeds such as Salers, Blonde d’Aquitaine or crossbreeds. Calves are weaned in France at pasture and later transferred to Italy for the fattening period. They arrive at the farm at approximately 10–14 months of age and 300 to 400 kg of weight, and are reared under intensive fattening conditions to reach slaughter weight after an extra period of about 6–7 months. Each farm can have a turnover of about two fattening cycles per year. The typical housing system consist of closed or open barns with multiple pens and fully slatted floors or concrete floors with bedding^44^. All fattening farms operate according to a quite standardized feeding management which supplies a high input in terms of feed. The diet is based on total mixed ration provided with a high proportion of concentrates, different proportions of feedstuffs according to genotype, gender and phase of the fattening cycle, and mineral and vitamin supplementations^45^. Wheat straw and soybean meal are the main sources of fibers and proteins, respectively, whereas corn silage is the principal component of the total mixed ration. Occasionally, dry or pressed ensiled sugar beet pulps are used as non-starchy energy feeds.

Beef cattle reared in intensive farming systems have to deal with several welfare issues during the fattening cycle. Main welfare problems are respiratory and digestive disorders such as the bovine respiratory disease and the sub-acute ruminal acidosis, leading to higher risk of chronic pneumonia, rumen parakeratosis and liver abscesses^11^. These are linked, for instance, to inadequate ventilation systems, inappropriate diets, overstocking and social mixing procedures. These risk factors combined with other potential stressful events/conditions (e.g. weaning, transportation, lack of enrichments or negative animal handling), make intensively reared beef cattle also more susceptible to the development of behavioural problems. Some examples are aggressive behaviour (e.g. fighting and chasing), unusual resting behaviour and fearfulness towards humans which can be a sign of discomfort^11^. Finally, other major detrimental issues in beef production are locomotor disorders such as lameness, bursitis and claw lesions, which mainly compromise the welfare of heavy breeds. Studies showed that the provision of appropriate straw/rubber-bedding areas may be beneficial for the reduction of these injuries^46^.

Therefore, this study aimed to investigate the impact of welfare and biosecurity on AMU in beef cattle. The hypothesis is that higher standards of welfare and biosecurity practices in beef fattening farms are associated to lower levels of AMU. This may become essential for the development of preventive on-farm strategies which in turn might lead to a more prudent antimicrobial stewardship.

## Materials and methods

### Data collection

Data used in this study were recorded in specialised Italian fattening farms located in Veneto region by a cooperative of beef producers (AZoVe, Cittadella, Italy) who are the owners of the animals, and covered the period from January 2016 to April 2019. All animals were first purchased from different beef breeding herds by French cattle cooperatives or private companies, and later sold to Italian beef producers. Data collected, initial editing procedure and calculation were performed as described by Diana et al.^47^. The dataset accounted for 1487 batches from 35 beef fattening farms and a total of 87 902 animals, with information on farm, sex, breed, body weight, date of beginning and end of the fattening cycle, number of animals and number of casualties. Percentage of animals treated, length of the fattening cycle (days), ADG and mortality rate were calculated per batch which constituted of animals of the same sex and breed. Data of those veterinary medicinal products (VMP, n = 33) containing antimicrobials, which were initially identified by the name of the product and later re-coded by assigning a number from 1 to 33, were recorded as well as the number of parenteral treatments administered to the animals during the study. A defined daily dose for animals was established for each active ingredient with antimicrobial activity of those VMP used in the study. Specifically, the defined daily dose for animals of a VMP represents the dose (in mg) of the active ingredient administered per kg of body weight per day. Finally, data on welfare standards and biosecurity were collected at farm level and matched by batch with all the aforesaid information.

Batches with missing data on welfare, biosecurity, body weight at the beginning and/or at the end of the fattening cycle as well as breeds with less than 20 batches and single-breed farms were discarded from the dataset. The final dataset included 1294 batches from 27 beef farms and seven beef breeds: Charolaise, Blonde d’Aquitaine, Limousine, Salers, Italian crossbred, French crossbred and Irish crossbred. A total of 175 124 parenteral treatments were administered to the animals during the whole period of study.

### Calculation of the treatment incidence 100

Calculation of the treatment incidence 100 (TI100) was also carried out as described by Diana et al.^47^. Defined daily dose for animals were used to calculate the TI100 which quantifies the frequency of treatment and allows for a better monitoring of AMU among countries in livestock farming^48,49^. Specifically, a defined daily dose for animals for Italy (DDDAit) was estimated by using Italian summaries of product characteristics which were established during the development of the ClassyFarm integrated monitoring system (www.classyfarm.it) of the Italian Ministry of Health. The following formula, modified from Timmerman et al.^19^, was used to calculate the treatment incidence 100 for Italy (TI100it) per each VMP^50^:

$${\text{TI1}}00{\text{it }} = \frac{{{\text{ Amount of active ingredient administered per batch }}\left( {{\text{mg}}} \right){ }}}{{{\text{DDDAit }}({\text{mg}}/{\text{kg}}/{\text{day}}) \times {\text{ Animals at risk }} \times {\text{ Standard weight }}\left( {{\text{kg}}} \right) \times {\text{ Days at risk}}}} \times 100$$

where ‘animals at risk’ identified the number of animals of the batch; ‘standard weight’ was the average body weight of animals at treatment (400 kg) and ‘days at risk’ is the standard number of days of the fattening cycle (230 days).

The TI100it of each VMP were summed to obtain a total TI100it per batch. A second TI100, namely TI100vet, was calculated using the same formula but replacing the DDDAit with the European Medicines Agency’s DDDvet^51^. However, some DDDvet were not included in the TI100vet calculation because they were not available for four active ingredients. Also, two more indexes (HPCIA TI100it and HCPIA TI100vet) were calculated in a similar manner but using only those VMP classified as ‘Highest Priority Critically Important Antimicrobials’ (HPCIA) because considered as key contributors for the development of AMR^52^.

### Welfare and biosecurity assessment

A welfare farm assessment was carried out once a year at least for 1 year over the period of study by using a modified version of the Italian protocol for the assessment of dairy cow welfare in loose housing systems^53^, which is part of the ClassyFarm monitoring scheme. The protocol was made by a list of 56 items holding information about indicators of animal health and welfare, farm management practices and housing systems (Supplementary Table S1). All items were grouped into three main sections: Total welfare (items from 1 to 42), Biosecurity (items from 43 to 52) and Emergency management (items from 53 to 56). Total welfare section was divided into three areas (Area A: farm management and staff training – items from 1 to 13; Area B: housing – items from 14 to 29; Area C: animal-based measures – items from 30 to 42). Data were collected by 6 trained veterinarians who assigned a score to each item based on a 3-point scale scoring system (from 1 to 3) where 1 indicated a high level of risk or else a poor status of the item assessed whereas 3 indicated a low risk or a better status of the item. It is important to highlight that each item had a different ‘weight’ according to its potential impact on animal health and welfare^53^, so that the score assigned was considered more or less significant in the calculation of the final value. A value for biosecurity, emergency management and each of the three areas of total welfare was calculated per farm by summing the score of those items belonging to each section/area. The value for the section total welfare was calculated as reported in Ginestreti et al.^54^, considering a 50% contribution by Areas A and B and the remaining 50% by Area C. All these values were then expressed into percentage from 0 to 100 where zero indicated a poor status (i.e. lack of any welfare/biosecurity measures) and 100 indicated a good status (i.e. full application of welfare/biosecurity measures). Data on total welfare, Areas A, B and C, biosecurity and emergency management were re-coded into three categories each as for the guidelines of the Italian welfare protocol: low, medium and high. Specifically, a value up to 59% corresponded to a poor status of the section (i.e. low), from 60 to 80% to a medium status (i.e. medium) and above 80% to a good status (i.e. high). Finally, data obtained from the welfare farm assessment were matched with data on AMU and all the aforesaid information by assigning to the batches of each farm the associated score calculated for each section.

### Statistical analysis

Data were analysed using SAS 9.4 (SAS Institute Inc., Cary, NC, USA) with batch as experimental unit and were checked for normality using the Shapiro–Wilk test, skewness and kurtosis, and visual inspection of the normal plot. Pearson correlations were used to investigate the relationships between the three sections of the on-farm assessment (i.e. total welfare, biosecurity and emergency management) and between the three areas of total welfare section (i.e. Area A, B and C). Results are presented as the correlation coefficient (Rho). An ANOVA test in GLM procedure of SAS was used to check for: 1) statistical differences among years and breeds for total welfare, biosecurity and emergency management scores; 2) statistical differences among categories of total welfare, biosecurity and emergency management for performance traits and length of the fattening cycle. All TI100 indexes were not normally distributed, thus a generalized linear mixed model with gamma distribution and log link function in GLIMMIX procedure of SAS was used to investigate whether welfare standards and biosecurity practices are sources of variation of the aforesaid indexes. Data on total welfare, Areas B and C, biosecurity and emergency management were available for two of the three categories (low, medium and high), whereby the missing category was not included in the final analysis. The model accounted for sex, season, total welfare (two categories: medium, high), biosecurity (two categories: low, medium), emergency management (two categories: low, high) and the interaction between total welfare and sex as fixed effects, and farm and breed as random effects. In addition, a second model was used to check the effect of each welfare area on the TI100 indexes by substituting the variable total welfare with Area A (three categories: low, medium, high), B (two categories: low, medium) and C (two categories: medium, high) as predictors. Results are presented as least squares means (LSM) ± standard error of the mean (SEM). A Tukey–Kramer adjustment was used to account for multiple comparisons. The criterion for determination of statistical significance was established at P < 0.05 while statistical trend was established at 0.05 > P < 0.10.

### Ethics approval

This study was approved by the Ethical Committee for the Care and Use of Experimental Animals of the University of Padova, Italy (approval no. 74/2018). The study was conducted in accordance with Italian law (Decreto legislativo no. 26/2014) and EU Directive 2010/63/EU on the protection of animals used for scientific purposes.

## Results

### Performance traits and length of the fattening cycle per categories of welfare, biosecurity and emergency management

The average farm had 596.8 heads (SD = 295.9). Average batch size for total welfare was 56.3 vs. 60.7 heads (categories high and medium, respectively), 30.9 vs. 60.6 heads for biosecurity (categories medium and low, respectively) and 35.7 vs. 60.4 heads for emergency management (categories high and low, respectively; Table 1). Percentage of animals treated was 63.5 vs. 64.8% (categories high and medium, respectively) for total welfare, 30.9 vs. 65.2% (high and low, respectively) for emergency management and 78.5 vs. 64.2% (medium and low, respectively) for biosecurity. Descriptive data of the 3 areas of total welfare are reported in Table 1. Performance traits and length of the fattening cycle were significantly different between categories of welfare, biosecurity and emergency management (P < 0.05) and between the 3 areas of total welfare (P < 0.05) except for the initial body weight between categories of biosecurity and ADG between categories of Area B (P > 0.05; Table 2). The lowest initial body weight was reported for the highest category of total welfare (337.4 vs. 354.5 kg for categories high and medium, respectively), biosecurity (333.8 vs. 351.7 kg for medium and low, respectively) and emergency management (324.8 vs. 351.8 kg for high and low, respectively). Similar patterns were observed for the final body weight between categories of total welfare and emergency management, but not for biosecurity where the lowest final body weight was observed for the category low (634.9 vs. 656.8 kg for low and medium, respectively). The number of days spent in the production cycle was lower for the highest category of total welfare (200.3 vs. 205.1 d for high and medium, respectively), while the opposite result was observed for biosecurity (213.0 vs. 203.9 d for medium and low, respectively) and emergency management (247.1 vs. 203.3 d for high and low, respectively; Table 2).

**Table 1:** Descriptive statistics of number of animals per batch, mortality rate, number and percentage of animals treated for each category (low, medium and high) of total welfare, Area A, Area B, Area C, biosecurity and emergency management.

Item	Category	Animals per batch (n)				Mortality rate (%)				Animals treated (n)				Animals treated (%)			
		Mean	SD	Min	Max	Mean	SD	Min	Max	Mean	SD	Min	Max	Mean	SD	Min	Max
Total welfare	Low (< 60%)	NA				NA				NA				NA			
	Medium (60% to 80%)	60.76	34.39	15.0	220.0	0.74	1.51	0.0	15.56	40.79	34.99	1.0	219.0	64.79	39.03	1.45	100
	High (> 80%)	56.35	36.25	15.0	170.0	0.46	1.37	0.0	11.11	34.80	34.55	1.0	150.0	63.46	39.35	1.32	100
Area A	Low (< 60%)	55.59	25.87	20.0	121.0	0.37	1.13	0.0	5.00	42.48	33.88	1.0	121.0	70.49	36.26	2.86	100
	Medium (60% to 80%)	57.49	34.06	15.0	220.0	0.71	1.48	0.0	12.77	39.74	34.32	1.0	219.0	67.13	38.70	1.45	100
	High (> 80%)	69.31	34.43	15.0	176.0	0.62	1.56	0.0	15.56	39.07	37.48	1.0	176.0	54.42	39.22	1.32	100
Area B	Low (< 60%)	58.91	35.09	15.0	220.0	0.71	1.58	0.0	15.56	38.51	35.08	1.0	219.0	63.82	39.46	1.45	100
	Medium (60% to 80%)	62.35	32.44	15.0	203.0	0.63	1.25	0.0	9.68	42.42	34.60	1.0	176.0	66.26	38.16	1.32	100
	High (> 80%)	NA				NA				NA				NA			
Area C	Low (< 60%)	NA				NA				NA				NA			
	Medium (60% to 80%)	60.08	35.34	15.0	220.0	1.09	1.69	0.0	9.68	37.72	32.27	1.0	210.0	61.19	38.42	2.86	100
	High (> 80%)	59.88	33.98	15.0	219.0	0.56	1.40	0.0	15.56	40.27	35.77	1.0	219.0	65.58	39.24	1.32	100
Biosecurity	Low (< 60%)	60.64	34.37	15.0	220.0	0.68	1.48	0.0	15.56	40.09	35.24	1.0	219.0	64.20	39.07	1.32	100
	Medium (60% to 80%)	30.87	10.71	15.0	56.0	0.86	1.92	0.0	6.67	22.32	12.20	2.0	54.0	78.53	37.53	6.67	100
	High (> 80%)	NA				NA				NA				NA			
Emergency management	Low (< 60%)	60.42	34.46	15.0	220.0	0.68	1.49	0.0	15.56	40.24	35.05	1.0	219.0	65.23	38.94	1.32	100
	Medium (60% to 80%)	NA				NA				NA				NA			
	High (> 80%)	35.69	4.99	28.0	50.0	0.89	1.36	0.0	3.03	11.35	12.28	1.0	50.0	30.90	30.46	2.86	100

NA, no batch felt within the category; SD, standard deviation.

**Table 2:** Means and standard deviation (SD) of performance traits and number of days spent in the fattening cycle for each category (low, medium and high) of total welfare, Area A, Area B, Area C, biosecurity and emergency management.

Item	Category	Average daily gain (kg/d)				Initial body weight (kg)				Final body weight (kg)				Length of fattening cycle (d)			
		Mean	SD	Min	Max	Mean	SD	Min	Max	Mean	SD	Min	Max	Mean	SD	Min	Max
Total welfare	Low (< 60%)	NA				NA				NA				NA			
	Medium (60% to 80%)	1.40	0.27	0.22	2.38	354.5	60.1	187.0	535.0	641.3	86.9	409.7	798.5	205.1	29.5	98.2	338.7
	High (> 80%)	1.37	0.28	0.39	1.80	337.4	58.4	222.0	630.1	610.7	95.8	395.1	786.9	200.3	21.0	90.6	307.0
Area A	Low (< 60%)	1.39	0.28	0.98	1.86	326.9	58.5	243.3	414.7	626.2	94.5	475.3	743.2	218.2	37.7	181.0	325.2
	Medium (60% to 80%)	1.36	0.28	0.22	2.38	346.4	58.8	187.0	630.1	625.1	90.8	395.1	798.5	205.3	28.6	90.6	338.7
	High (> 80%)	1.51	0.19	0.80	2.07	372.1	60.2	237.0	520.4	674.5	71.8	469.5	786.9	198.6	24.4	122.3	280.4
Area B	Low (< 60%)	1.39	0.27	0.22	2.38	357.4	61.3	187.0	535.0	643.5	90.1	395.1	798.5	205.8	29.7	98.2	338.7
	Medium (60% to 80%)	1.39	0.26	0.34	1.89	336.8	54.5	203.9	630.1	616.5	85.3	409.7	785.1	200.3	23.7	90.6	316.9
	High (> 80%)	NA				NA				NA				NA			
Area C	Low (< 60%)	NA				NA				NA				NA			
	Medium (60% to 80%)	1.41	0.27	0.34	2.07	355.3	65.1	211.0	520.4	638.3	85.1	409.7	788.9	199.3	27.3	122.3	298.3
	High (> 80%)	1.39	0.27	0.22	2.38	350.5	58.5	187.0	630.1	634.6	90.5	395.1	798.5	205.7	28.3	90.6	338.7
Biosecurity	Low (< 60%)	1.39	0.27	0.22	2.38	351.7	60.1	187.0	630.1	634.9	89.7	395.1	798.5	203.9	28.2	90.6	338.7
	Medium (60% to 80%)	1.52	0.18	0.80	1.78	333.8	61.5	248.0	492.0	656.8	77.3	469.5	786.9	213.0	23.8	156.5	261.2
	High (> 80%)	NA				NA				NA				NA			
Emergency management	Low (< 60%)	1.40	0.26	0.22	2.38	351.8	60.6	187.0	630.1	637.2	89.4	395.1	798.5	203.3	27.6	90.6	338.7
	Medium (60% to 80%)	NA				NA				NA				NA			
	High (> 80%)	0.89	0.15	0.34	1.09	324.8	12.1	301.0	348.0	550.6	34.2	477.3	600.5	247.1	24.1	197.7	284.4

NA = no batch felt within the category.

### Total welfare, biosecurity and emergency management data

**Figure 1:**
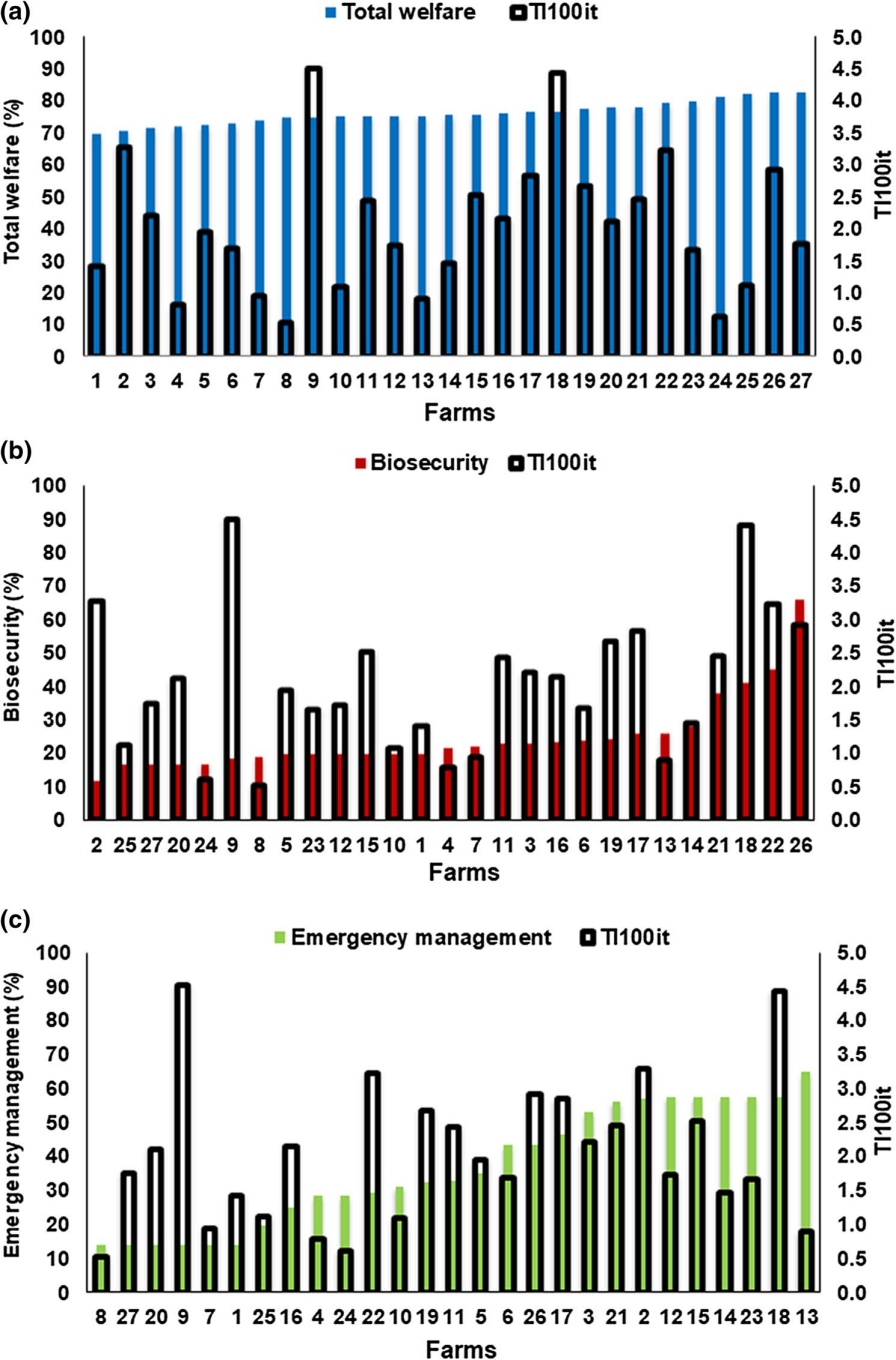
Descriptive statistics of the score of (**a**) total welfare, (**b**) biosecurity, (**c**) emergency management and TI100it^1^ of beef farms included in the study (n = 27).^1^TI100it = treatment incidence 100 for Italy, calculated by using the defined daily dose for animals for Italy based on Italian guidelines of dosage obtained from the Italian database (www.classyfarm.it).

Overall, 59.3% of the farms were assessed for 3 years, 22.2% of the farms for 2 years and 18.5% of the farms for 1 year. Total welfare per batch ranged from 66 to 84% (mean = 76%, SD = 5%), biosecurity ranged from 9 to 66% (mean = 24%, SD = 12%) and emergency management from 14 to 83% (mean = 39%, SD = 20%). Data obtained for the 3 areas of total welfare ranged from 59 to 86% (mean = 74%, SD = 6%) for Area A (farm management and staff training), 40 to 75% (mean = 56%, SD = 8%) for Area B (housing) and 63 to 100% (mean = 87%, SD = 9%) for Area C (animal-based measures). Specifically, a considerable variability was observed between the 27 farms for biosecurity and emergency management (Fig. 1).

**Figure 2:**
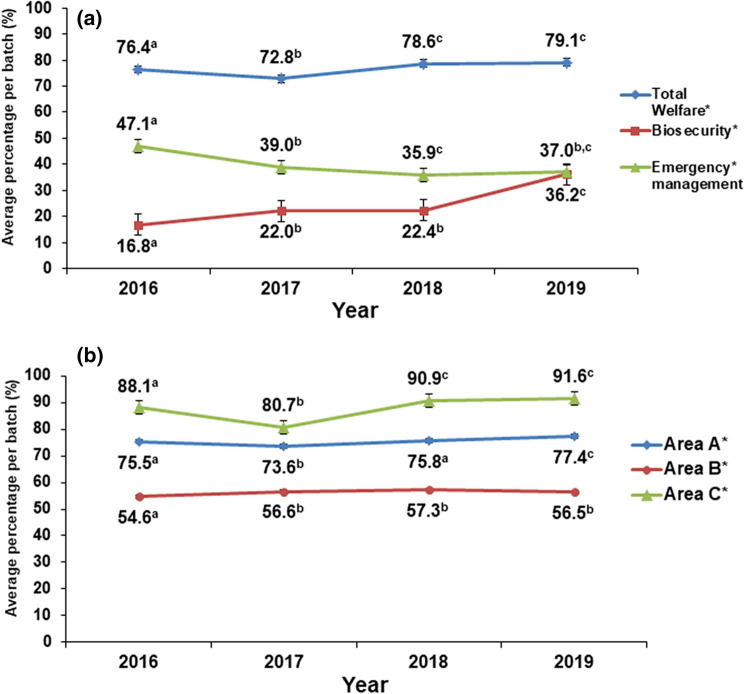
Average percentage (%) of the score of (**a**) total welfare, biosecurity and emergency management and of (**b**) Area A, Area B and Area C of total welfare among years in beef cattle.^a,b^Different superscript letters within each sector (e.g. total welfare, biosecurity and emergency management) and area (Area A, B and C) indicate significant differences (P < 0.05).^*^P < 0.001.

Correlations between total welfare, biosecurity and emergency management and between the 3 areas of total welfare are presented in Table 3. Specifically, total welfare and biosecurity were positively correlated (Rho = 0.31) while low correlations were found for total welfare and emergency management (Rho = -0.17) and for biosecurity and emergency management (R = 0.20). Total welfare, biosecurity and emergency management scores were significantly different among years (P < 0.001; Fig. 2) and breeds (P < 0.01; Table 4) except for biosecurity that tended to be different among breeds (P = 0.079).

**Table 3:** Pearson correlations between the average score (%) of total animal welfare, biosecurity, emergency management and the 3 areas of total welfare (Area A, B and C) in beef cattle.

Item	1	2	3	4	5
Total welfare, 1					
Biosecurity, 2	0.31**				
Emergency management, 3	− 0.17**	0.20**			
Area A, 4	0.65**	0.29**	− 0.11*		
Area B, 5	0.53*	0.19**	− 0.06*	− 0.11**	
Area C, 6	0.88**	0.17**	− 0.13**	0.44**	− 0.35*

^1^Total welfare = this section consists of variables grouped and listed within Area A, Area B and Area C (Supplementary Table S1).

^2^Biosecurity = some examples of the variables included are control of visitors, quarantine, control of rodents and lorry cleaning (Supplementary Table S1).

^3^Emergency management = variables included are fire alarm, ventilation alarm, risk of noise and source of drinking water (Supplementary Table S1).

^4^Area A = farm management and staff training (e.g. feeding, cleaning, n. of employees, n. of animal inspections; Supplementary Table S1).

^5^Area B = housing (e.g. flooring system, lighting system, hospital pen; Supplementary Table S1).

^6^Area C = animal-based measures (e.g. respiratory disease, human-animal interaction, aggressive behaviour, lesions, lameness; Supplementary Table S1).

*P < 0.05; **P < 0.001.

**Table 4:** Least squares means (LSM) and standard error of the mean (SEM) of the average score (%) of total welfare, biosecurity, emergency management and the 3 areas of total welfare (Area A, B and C) by breeds^1^ in beef cattle.

Item	BDA		CHR		FRC		IRC		ITC		LIM		SAL		P value
	LSM	SEM	LSM	SEM	LSM	SEM	LSM	SEM	LSM	SEM	LSM	SEM	LSM	SEM	
Total welfare^2^	77.78^b,d^	0.93	77.08^c,d^	0.18	75.83^a,b^	0.47	77.75^c,d^	0.84	75.24^a^	0.49	76.79^b,c^	0.27	76.81^a,b,d^	0.90	0.003
Area A^3^	76.60^b,c,d^	1.33	75.22^b,c^	0.26	74.92^a,b^	0.68	75.38^a,c^	1.20	73.21^a^	0.70	73.63^a^	0.38	80.05^d^	1.29	< 0.001
Area B^4^	60.11^c,d^	1.65	55.37^b^	0.32	51.15^a^	0.84	55.92^b,c^	1.49	54.73^b^	0.87	58.47^c,d^	0.47	58.08^b,d^	1.60	< 0.001
Area C^5^	87.41^a,c^	1.65	89.12^c^	0.32	88.92^b,c,d^	0.84	90.08^c,d^	1.49	86.74^a,b^	0.87	87.74^a,d^	0.47	84.83^a^	1.60	0.005
Biosecurity^6^	23.74^a,b^	2.53	26.28^b,c,d,e^	0.49	24.46^a,d^	1.29	24.87^a,e^	2.29	24.35^a,c^	1.34	26.62^b,c,d,e^	0.73	20.06^a^	2.45	0.079
Emergency^7^ management	56.24^e^	4.26	39.44^b^	0.83	44.76^c,d^	2.17	39.16^b,c^	3.85	39.38^b,d^	2.25	38.18^b^	1.22	21.09^a^	4.13	< 0.001

^a,b^Different superscript letters between breeds within each sector or area indicate significant differences (P < 0.05).

^1^BDA = Blonde d’Aquitaine, CHR = Charolaise, FRC = French crossbred, IRC = Irish crossbred, ITC = Italian crossbred, LIM = Limousine, SAL = Salers.

^2^Total welfare = this section consists of variables grouped and listed within Area A, Area B and Area C (Supplementary Table S1).

^3^Area A = farm management and staff training (e.g. feeding, cleaning, n. of employees, n. of animal inspections; Supplementary Table S1).

^4^Area B = housing (e.g. flooring system, lighting system, hospital pen; Supplementary Table S1).

^5^Area C = animal-based measures (e.g. respiratory disease, human-animal interaction, aggressive behaviour, lesions, lameness; Supplementary Table S1).

^6^Biosecurity = some examples of the variables included are control of visitors, quarantine, control of rodents and lorry cleaning (Supplementary Table S1).^7^Emergency management = variables included are fire alarm, ventilation alarm, risk of noise and source of drinking water (Supplementary Table S1).

### Effect of welfare, biosecurity and emergency management on the treatment incidence

Antimicrobial use differed significantly between categories of total welfare. Specifically, lower AMU was observed with higher welfare conditions (> 80%) for TI100it (1.25 vs. 1.61 for high and medium categories, respectively; P = 0.008) and TI100vet (1.02 vs. 1.28 for high and medium, respectively; P = 0.021) whereas, despite a similar trend, no differences were reported for the corresponding HPCIA indexes (P > 0.05; Table 5). No significant differences were reported between categories of biosecurity and emergency management for the investigated TI100 indexes.

**Table 5:** Least squares means (LSM) and standard error of the mean (SEM) of the treatment incidence (TI100)^1^ for the effects of total welfare, biosecurity and emergency management in beef cattle.

Effect	Category	TI100it		TI100vet		HPCIA TI100it		HPCIA TI100vet	
		LSM	SEM	LSM	SEM	LSM	SEM	LSM	SEM
Total welfare^2^	Low (< 60%)	NA	NA	NA	NA	NA	NA	NA	NA
	Medium (60% to 80%)	1.61^a^	0.45	1.28^a^	0.42	0.98^a^	0.34	0.89^a^	0.37
	High (> 80%)	1.25^b^	0.35	1.02^b^	0.34	0.81^a^	0.28	0.75^a^	0.32
Biosecurity^3^	Low (< 60%)	1.24^a^	0.23	0.87^a^	0.17	0.71^a^	0.17	0.58^a^	0.14
	Medium (60% to 80%)	1.63^a^	0.76	1.51^a^	0.87	1.12^a^	0.65	1.14^a^	0.88
	High (> 80%)	NA	NA	NA	NA	NA	NA	NA	NA
Emergency management^4^	Low (< 60%)	1.62^a^	0.42	1.31^a^	0.40	1.05^a^	0.34	0.94^a^	0.38
	Medium (60% to 80%)	NA	NA	NA	NA	NA	NA	NA	NA
	High (> 80%)	1.25^a^	0.40	1.00^a^	0.36	0.76^a^	0.30	0.71^a^	0.33

^a,b^Different superscript letters within each TI100 index and effect indicate significant differences (P < 0.05); NA = no batch felt within the category.

^1^TI100it = treatment incidence 100 for Italy, calculated by using the defined daily dose for animals for Italy based on Italian guidelines of dosage obtained from the Italian database (http://www.classyfarm.it); TI100vet = treatment incidence 100 for EU, calculated by using the defined daily dose for animals for Europe based on EMA’ guidelines of dosage^51^; HPCIA = Highest Priority Critically Important Antimicrobials.

^2^Total welfare = this section consists of variables grouped and listed within Area A, Area B and Area C (Supplementary Table S1).

^3^Biosecurity = some examples of the variables included are control of visitors, quarantine, control of rodents and lorry cleaning (Supplementary Table S1).^4^Emergency management = variables included are fire alarm, ventilation alarm, risk of noise and source of drinking water (Supplementary Table S1).

No significant differences were also observed between categories of Area A, Area B and Area C of total welfare for the TI100it and the TI100vet (P > 0.05; Table 6). No significant differences between categories of Area A, Area B and Area C of total welfare were also observed for the HPCIA TI100it (P > 0.05), whereas lower AMU was observed with higher welfare in Area A only for the HPCIA TI100vet (P = 0.034; Table 6).

**Table 6:** Least squares means (LSM) and standard error of the mean (SEM) of the treatment incidence (TI100)^1^ for the effects of Area A, Area B and Area C in beef cattle.

Effect	Category	TI100it		TI100vet		HPCIA TI100it		HPCIA TI100vet	
		LSM	SEM	LSM	SEM	LSM	SEM	LSM	SEM
Area A^2^	Low (< 60%)	1.38^a^	0.48	1.16^a^	0.43	1.05^a^	0.45	1.21^a^	0.57
	Medium (60% to 80%)	1.46^a^	0.42	1.25^a^	0.39	0.95^a^	0.33	0.98^a^	0.40
	High (> 80%)	1.42^a^	0.40	1.07^a^	0.33	0.82^a^	0.28	0.77^b^	0.31
Area B^3^	Low (< 60%)	1.37^a^	0.41	1.09^a^	0.36	0.89^a^	0.32	0.91^a^	0.38
	Medium (60% to 80%)	1.47^a^	0.44	1.23^a^	0.39	0.98^a^	0.35	1.04^a^	0.43
	High (> 80%)	NA	NA	NA	NA	NA	NA	NA	NA
Area C^4^	Low (< 60%)	NA	NA	NA	NA	NA	NA	NA	NA
	Medium (60% to 80%)	1.48^a^	0.44	1.17^a^	0.38	0.94^a^	0.33	0.99^a^	0.41
	High (> 80%)	1.36^a^	0.40	1.14^a^	0.37	0.93^a^	0.33	0.95^a^	0.40

^a,b^Different superscript letters within each TI100 index and effect indicate significant differences (P < 0.05); NA = no batch felt within the category.

^1^TI100it = treatment incidence 100 for Italy, calculated by using the defined daily dose for animals for Italy based on Italian guidelines of dosage obtained from the Italian database (www.classyfarm.it); TI100vet = treatment incidence 100 for EU, calculated by using the defined daily dose for animals for Europe based on EMA’ guidelines of dosage^51^; HPCIA = Highest Priority Critically Important Antimicrobials.

^2^Area A = farm management and staff training (e.g. feeding, cleaning, n. of employees, n. of animal inspections; Supplementary Table S1).

^3^Area B = housing (e.g. flooring system, lighting system, hospital pen; Supplementary Table S1).

^4^Area C = animal-based measures (e.g. respiratory disease, human-animal interaction, aggressive behaviour, lesions, lameness; Supplementary Table S1).

Finally, breeds tended to be different for all TI100 indexes: TI100it (0.119 ± 0.073; P = 0.052), TI100vet (0.113 ± 0.073; P = 0.061), HPCIA TI100it (0.194 ± 0.123; P = 0.058) and HPCIA TI100vet (0.118 ± 0.082; P = 0.074) indicating a substantial variability of AMU among breeds (Table 7).

**Table 7:** Estimate and standard error (SE) of the treatment incidences (TI100)^a^ for the effect of breed^b^ in beef cattle.

Breed	TI100it		P value	TI100vet		P value	HPCIA TI100it		P value	HPCIA TI100vet		P value
	Estimate	SE		Estimate	SE		Estimate	SE		Estimate	SE	
Intercept	0.119	0.073	0.052	0.113	0.073	0.061	0.194	0.123	0.058	0.118	0.082	0.074
BDA	0.395^^^	0.217		0.438*	0.219		0.545*	0.271		0.401^^^	0.242	
CHR	− 0.173	0.143		− 0.038	0.140		− 0.020	0.183		− 0.116	0.147	
FRC	− 0.144	0.162		− 0.109	0.158		− 0.093	0.204		− 0.027	0.171	
IRC	− 0.202	0.194		− 0.274	0.195		− 0.482^^^	0.257		− 0.359	0.228	
ITC	− 0.544*	0.164		− 0.404*	0.159		− 0.500*	0.207		− 0.348*	0.176	
LIM	0.434*	0.150		0.441*	0.147		0.592*	0.192		0.453*	0.159	
SAL	0.032	0.212		− 0.098	0.216		− 0.123	0.277		− 0.049	0.238	

^a^TI100it = treatment incidence 100 for Italy, calculated by using the defined daily dose for animals for Italy based on Italian guidelines of dosage obtained from the Italian database (http://www.classyfarm.it); TI100vet = treatment incidence 100 for EU, calculated by using the defined daily dose for animals for Europe based on EMA’ guidelines of dosage^51^; HPCIA = Highest Priority Critically Important Antimicrobials.

^b^BDA = Blonde d’Aquitaine, CHR = Charolaise, FRC = French crossbred, IRC = Irish crossbred, ITC = Italian crossbred, LIM = Limousine, SAL = Salers.

Statistically different from the intercept = ^^^0.10 < P < 0.05; *P < 0.05.

## Discussion

The objective of this study was to investigate whether some representative farm factors such as welfare standards and biosecurity practices may be identified as sources of variation of AMU in beef cattle. Previous studies reported that typical farm factors such as management practices, farm size, feeding and housing systems or tailored welfare-friendly procedures are likely to affect AMU^24,35,36,55,56^. Therefore, the variability of levels of total welfare, biosecurity and emergency management reported among farms may contribute to justify the different AMU observed between and within Italian beef farms as described in our previous work^27,47^. Specifically, a significant reduction of frequency of treatment was observed with improved level of welfare making it a potential source of variation of AMU in beef cattle, while no significant results were detected for biosecurity and emergency management.

Reduction of antimicrobials can be achieved if animal health and welfare are not compromised. Hence, it is fundamental to measure parameters considered as reliable indicators of animal health and welfare when running studies on AMU. On average, a high score (76%) was observed for ‘total welfare’ among farms indicating a great attention of farmers towards this component for an efficient livestock management^12,57^. Moreover, lower AMU was linked to those batches with improved total welfare conditions, which is in accordance with other studies^24,42,58^. Given the relationship between animal health and welfare^3,4^, the significant lower AMU observed with a high level of welfare made us argue that those batches were also likely to experience an improved health status. Indeed, some of the animal-based measures listed in the Area C of the section total welfare, were used to assess the presence or absence of health indicators such as lameness, lesions and respiratory diseases. Albeit not significant, a lower TI100it value was found with higher level of Area C, thus supporting our assumption that reduced AMU was associated to improved animal health. Similar results were observed by Becker et al.^42^ who found better animal health parameters in farms that followed an improved welfare fattening system, namely ‘outdoor veal calf’, compared with conventional fattening systems. The authors suggested a reduced AMU as animals were healthier.

Data were also analysed to explore the potential impact of each area of the section ‘total welfare’ on AMU. The results showed that, despite lower TI100it values were observed with higher level of Area A and C—while the opposite was reported for Area B—these differences were not significant. Hence, we may argue that significant values of TI100 can be detected only when data are combined together, as also suggested by Damiaans et al.^59^ in their study on biosecurity in cattle. They found that total biosecurity score was significantly higher in dairy than beef cattle. However, when single subcategories of biosecurity were tested, only two of them gave significant results (e.g. feed and water and calf management). The authors suggested that while the differences were too small to be significant for each distinct subcategory, the latter revealed their ‘hidden difference’ only through the calculation of the total biosecurity score. In practice, this may imply that a significant reduction of AMU is possible when all welfare standards of all areas are properly applied on-farm.

Findings from the current study also showed lower ADG and body weight with better welfare conditions. This is in accordance with the study of Becker et al.^42^ who reported a lower ADG (1.29 vs. 1.35 kg/d) and dry matter intake, albeit not significant, for veal calves reared under better welfare conditions. The authors explained the resulting lighter carcass weight of these calves either due to a different feeding strategy or to a colder air temperature because they were reared outdoor. Hence, it is likely that the higher energy requirement needed for thermoregulation, contributed to the lower ADG observed for calves reared with improved welfare standards.

High growth rate does not necessary mean that animals are equally experiencing good welfare^41^, emphasising the multifactorial nature of animal welfare^60,61^. This may contribute to further clarify the lower ADG/body weight observed with better welfare conditions and reported in our study. Indeed, the concept of animal welfare goes beyond disease conditions, reproductive health and growth rate, thus implying that only if reproduction or survival are compromised, the welfare of the animals is considered poor^62,63^. Animal welfare is more than that, it is also related to the animal’s emotional/mental state. According to the definition given by the World Organisation for Animal Health Terrestrial Code to animal welfare^1^, both the biological functioning and the natural behavioural needs are equally important to evaluate the physical and mental state of an animal^2^.

At first sight, low body weight may appear as a disadvantage of raising beef cattle with high welfare standards. However, potential economic loss that farmers may encounter with a reduced slaughter weight can be balanced by a reduction of antimicrobials cost^42^. Also, targeted-feeding programmes may be a valuable strategy to minimise such a loss. Future research should better investigate this relationship, as the impact of welfare standards and other farm-related variables on performance traits was not the objective of this study.

Statistically significant differences among breeds were observed for AMU, as also reported in our previous study^27^ where breed was identified as an important source of variation of AMU. In addition, results from the current study showed that breeds differed significantly for the score assigned to total welfare. Surprisingly, breeds with high welfare scores were not always those showing lower AMU. It is likely that certain welfare measures may result more/less effective according to the breed, like seen in the study of Magrin et al.^46^ on beef cattle. The authors reported that rubber-covered slatted floors helped to reduce the percentage of animals treated and culled for locomotor disorders only for Limousine breed. In fact, this type of welfare-friendly flooring system was not equally efficient for Charolaise beef breed, likely due to animals’ higher weight. This suggests that in order to maximise the positive effect of high welfare standards on AMU, tailored welfare measures must be implemented according to the breed(s) reared on-farm.

The efficacy of biosecurity measures in preventing diseases and improving animal health is widely documented^10,34,64^. Nonetheless, knowledge on the overall level of biosecurity in beef fattening farms is still poorly available and it seems difficult to implement. Data from this study described, for the first time, the level of biosecurity applied on Italian beef fattening farms suggesting room for improvement. Indeed, not only our findings reported a general low level of biosecurity among farms, with a mean score per batch of 24%, but also we realised that none of the batches scored > 80%—namely as ‘high’- which is the category indicating farms with the best conditions of biosecurity. This is in accordance with other studies where despite a general awareness on the importance of biosecurity in ensuring good animal health and performance, it was poorly implemented on-farm^54,59,65^. Damiaans et al.^59^ described a low mean biosecurity score in Belgian cattle farms with specifically veal (39.7) and beef (44.3) scoring lower than dairy (48.6) out of 100 points. Another study on Italian dairy cattle also reported similar results with a mean biosecurity score of 45.8% out of 100%^54^. It is possible that farmers are not willing to implement biosecurity measures especially when these are considered costly to apply or if the herd is not experiencing any performance/reproductive loss^65–67^. Also, providing ‘easy-to-access’ and concrete information on biosecurity levels, which are now lacking for beef cattle, will enable farmers to apply more accurate measures^64,68^. Similar results were also observed for the level of emergency management with the majority of the batches scoring lower than 60%—namely as ‘low’—and only 2.1% of them scoring more than 80% while no data were available for the category ‘medium’. This likely indicates that Italian beef fattening farms are not adequately prepared to deal with unexpected emergencies such as fires or disruption of the drinking system in the herd.

The general low scores reported in this study may have biased our findings because those farms reporting low AMU were also identified within the category of ‘low level of biosecurity’. Therefore, the lack of significant effect found for both biosecurity and emergency management sectors on AMU can likely be justified by the limited sample size of certain categories (i.e. ‘medium’ and ‘high’) with a bias of data towards lower scores. Such a shortage of data may also justify the numerically higher TI100 values, albeit not significant, observed with higher biosecurity which is somehow surprising. In fact, different studies showed that increased biosecurity was often associated to lower AMU and better health and welfare conditions of the animals^32,34,42^, thus supporting the idea that improved biosecurity should be a core objective of animal production. The positive correlation found in our study between biosecurity and welfare standards, somehow would strengthen this notion. However, there are also studies that observed few contradictions such as in Pandolfi et al.^5^ who reported a negative impact on mortality, prevalence of lameness and need of more hospitalizations in pigs belonging to the cluster with higher biosecurity score. Whereas, Calderón Díaz et al.^69^ reported that improved levels of certain biosecurity practices related to cleaning and disinfection (e.g. footbaths) were associated to an increased likelihood of PRRS disease. The authors explained the results suggesting that these measures were likely applied a posteriori rather than in a ‘preventive’ manner. Hence, further research is needed to better clarify this relationship in beef cattle too.

Finally, it is worth to highlight that the assessment of biosecurity was made by scoring those items that are identified as ‘external biosecurity’^8,59^. Indeed, it is likely that a significant reduction of AMU with improved level of biosecurity may be detected only by combining data of both external and internal biosecurity, as observed for the results of total welfare. Laanen et al.^33^ reported a significantly lower treatment incidence only when internal biosecurity was higher, leading in turn to significant results also for the overall biosecurity. According to literature, internal biosecurity seems to play an important role for the reduction of AMU^5,33,59^. For instance, within the practices classified as internal biosecurity, those related to the ‘health management’ such as isolation of sick animals or using of specific boots/clothes for hospital pens only, are essential to avoid contact between healthy and infected animals^59^. The latter, are recognised as major reservoir of infectious diseases^70,71^. This emphasises not only the crucial influence that single ‘internal’ practices may have on the overall biosecurity score^5,33^, but also the importance of combining internal and external biosecurity measures to obtain a more reliable information.

With this study, we also highlighted both the importance of defining data for farm benchmarking to be used in future research on AMU and the potential limitations of retrospective studies in obtaining reliable information on AMU. Indeed, these studies can have missing values and lack of a specific study design, thus requiring multiple data from different sources or the collection of data in different ways^5^. This was the case of the current study, where data on AMU were available at batch level while animal welfare and biosecurity assessments were made at farm level, whereby potentially explaining the lack of significant results between categories of both biosecurity and emergency management.

## Conclusion

In summary, a wide variation among farms for biosecurity and emergency management was reported. Specifically, while on average a high score (76%) was observed for total welfare indicating great attention of farmers towards this component, the level of biosecurity and emergency management in beef cattle was quite low (24 and 39%, respectively) suggesting room for improvement. Moreover, a significant reduction of AMU was observed with improved level of animal welfare. On the opposite, no significant effect on AMU was observed for biosecurity and emergency management likely due to a bias of data towards lower scores. These results may help to define benchmark data and to address the reduction of AMU in beef industry. For instance, we recommend major focus on strategies to improve both external and internal biosecurity or else targeted animal welfare programmes to satisfy the needs of each farm individually. Further research is still needed to confirm our findings. In fact, data coming from retrospective studies may only provide part of the story, whereas future longitudinal controlled studies will better clarify the relationship between welfare standards, biosecurity practices and AMU by removing other potential confounding factors.

## Supplementary information

Supplementary Information.

## Data Availability

The datasets generated and/or analysed during the current study are not publicly available due to their sensitive nature but are available from the corresponding author on reasonable request.
