# Supplementary material for: Effect of welfare standards and biosecurity practices on antimicrobial use in beef cattle
Source: Sci Rep. 2020 Dec 1;10:20939. doi: 10.1038/s41598-020-77838-w (PMC7708642; doi:10.1038/s41598-020-77838-w)
Supplement: Supplementary file 1 — Supplementary Information. [file 41598_2020_77838_MOESM1_ESM.pdf]

Supplementary information for:

Effect of welfare standards and biosecurity practices on antimicrobial use in beef cattle

Alessia Diana[1,*], Valentina Lorenzi[2], Mauro Penasa[1], Edoardo Magni[2], Giovanni L. Alborali[2], Luigi Bertocchi[2] and Massimo De Marchi[1]

[1]Department of Agronomy, Food, Natural resources, Animals and Environment (DAFNAE), University of Padova, Viale dell'Università 16, 35020 Legnaro (PD), Italy

[2]Italian National Reference Centre for Animal Welfare, Istituto Zooprofilattico Sperimentale della Lombardia e dell'Emilia Romagna 'Bruno Ubertini' (IZSLER), Via Bianchi 9, 25124 Brescia, Italy

[*]Corresponding author: alessiadiana84@gmail.com

Table S1. Welfare and biosecurity assessment protocol used on the studied beef farms (n=27) and modified from the Italian protocol for the assessment of dairy cow welfare in loose housing systems [53][1] which is part of the ClassyFarm integrated monitoring system (www.classyfarm.it).

**SECTION - ANIMAL WELFARE**			
**Area A - Farm management and staff training**			
N	***Item***	***Risk level***	
1	Number of stockpersons	1 stockman for ≥ 800 animals	1
		1 stockman for 400-800 animals	2
		1 stockman for ≤ 400 animals	3
2	Experience and training of stockpersons	experience ≤ 10 years and no training courses on beef cattle farming during the last 3 years	1
		experience ≥ 10 years and no training course on beef cattle farming during the last 3 years	2
		experience ≥ 10 years and appropriate qualification or attendance to a recognized training course on beef cattle farming during the last 3 years	3
3	Group size and grouping strategy	≥ 40 animals per group, heterogeneous in terms of animal body-weight, sex or age	1
		20-40 animals per group, homogeneous in terms of animal body-weight, sex or age	2
		≤ 20 animals per group, homogeneous in terms of body-weight, sex or age	3
4	Inspection of the animals	< 1 inspection/day	1
		1 or more than 1 inspection/day	2
		More than 1 inspection/day and registration of the events	3
5	Type of animal handling	use of harmful tools (e.g. electric goads or sharp tools)	1
		use of non-harmful tools (e.g. calm voice, hands and/or plastic poles)	2
6	Feeding strategy	empirical diet that do not meet the metabolic requirements of the animals	1
		diet calculated by a nutritionist and constantly up-dated	2
7	Feeding management according to the production phase	only 1 feeding phase despite the different production stages	1
		2 feeding phases (adaptation and growing)	2
		3 feeding phases (e.g. adaptation, growing and finishing)	3
8	Feed availability	feed not available for 24h	1
		separate ration correctly managed (concentrates administered at least 2 times/day) and available for 24h	2
		total mixed ration available for 24h	3
9	Concentrate feed (daily dose)	concentrate > 80% of the total dry matter intake and average content of fibre < 6%	1
		concentrate equal to 70%-80% of the total dry matter intake and average content of fibre ≥ 6%	2
		concentrate < 70% of the total dry matter intake and at least 1 kg of straw or hay	3
10	Water provision	1 or more animals without access to drinking water	1
		all animals have access to functioning drinking bowls	2
		all animals have access to functioning water troughs	3
11	Cleanliness of water points	both drinkers and water are dirty	1

		there are feed residues in the drinkers but water is fresh and clean	2
		both drinkers and water are clean	3
12	Cleanliness and management of the pens	the floor, the lying area and the bedding material are dirty and not managed in most of the pens	1
		the floor, the lying area and the bedding material are sufficiently clean and managed in most of the pens	2
		the area is correctly managed, the floor is clean and dry and the bedding material is frequently topped up in all the pens	3
13	Biosecurity	no biosecurity programme or inadequate biosecurity measures (i.e. score of the biosecurity section ≤ 33%)	1
		presence of minor biosecurity measures (i.e. score of the biosecurity section between 33.1% and 66%)	2
		presence of an effective biosecurity programme with an accurate recording system and availability of supporting documentation (i.e. score of the biosecurity section ≥ 66.1%)	3
**Area B – Housing**			
N	***Item***	***Risk level***	
14	Housing system	at least a group of animals is tied or it is kept untethered but with nothing to protect them from adverse climatic conditions (e.g. shelters)	1
		all animals are loose housed and protected from adverse climatic conditions	2
		all animals are loose housed with adequate shelter and they can have access either to an outdoor area (loafing area) or to a pasture	3
15	Space availability in the lying area (m^2/animal)	less than 2.5 m^2/head for animals up to 400 kg (plus 0.5 m^2/head per each extra 100 kg between 400 kg and 800 kg)	1
		between 2.5 and 4.5 m^2/head for animals up to 400 kg (plus 0.5 m^2/head per each extra 100 kg between 400 kg and 800 kg)	2
		more than 4.5 m^2/head for up to 400 kg (plus 0.5 m^2/head per each extra 100 kg between 400 kg and 800 kg)	3
16	Pen flooring system	presence of smooth and slippery floor (either slatted or solid)	1
		presence of slatted floor covered by rubber or presence of a non-slip solid floor or presence of deep litter with a small amount of bedding material	2
		presence of deep litter with optimal bedding material (plentiful, non-abrasive, well preserved, absorbent)	3
17	Electric trainers	use of electric trainers	1
		absence of electric trainers	2
18	Available space at feed bunk	in case of separate ration, number of feeding places < 100% of the total number of animals or, in case of total mixed ration, number of feeding places < 70% of the total number of animals	1
		in case of separate ration, as many feeding places as animals or, in case of total mixed ration, number of feeding places ≥ 70% of the total number of animals	2
		number of feeding places ≥ 120% of the total number of animals, suitable size and no risk of suffocation	3
19	Functioning and number of water points	less than 1 functioning water bowl for 13 animals or less than 6 cm of trough per animal	1
		1 functioning water bowl for 13 animals or 6 cm of trough per animal	2

		more than 1 functioning water bowl for 13 animals or more than 6 cm of trough per animal and different water access points	3
20	Bedding material for new-born calves (less than 2 weeks of age)	no or scarce bedding material	1
		presence of bedding material that covers the whole floor	2
21	Space availability for calves up to 8 weeks of age (in single pen)	pen size non-compliant with the minimum national legislative limits (D. Lgs. N.126/2011) or there is at least 1 calf tethered	1
		pen size compliant with the minimum national legislative limits (D. Lgs. N.126/2011)	2
		pen size greater than the minimum national legislative limits (at least > 10%)	3
22	Possibility for calves allocated in single pens, to see and touch each other	no	1
		yes	2
23	Space availability for calves in group pen	pen size non-compliant with the minimum national legislative limits (D. Lgs. N.126/2011) or there are calves, older than 8 weeks of age, housed in single pens or there is at least 1 calf tethered	
		pen size compliant with the minimum national legislative limits (D. Lgs. N.126/2011)	
		pen size greater than the minimum national legislative limits (at least > 10%)	
24	Handling facilities	lack of handling facilities	1
		presence and use of permanent handling facilities with open side panels	2
		presence and use of adjustable handling facilities with solid side panels	3
25	Restraining facilities (crushes or cages)	lack of restraining facilities	1
		presence of non-specific but effective facilities for immobilization of cattle	2
		presence of proper restraining facilities, specific for immobilization of cattle	3
26	Facilities for sick animals	lack of facilities for sick animals or presence of sick pens with fully slatted floors (no bedding)	1
		presence of facilities for sick animals provided with deep litter	2
		presence of specific facilities for sick animals which prevent the contact with healthy animals and provided with clean deep litter	3
27	Temperature, humidity and ventilation	inadequate temperature and humidity, or insufficient air movement, or closed buildings	1
		adequate temperature and humidity and sufficient natural air movement or ventilation, but ventilation does not have an automatic control system	2
		adequate temperature and humidity, thanks to an automatic ventilation or cooling system; otherwise summer pasture equipped with shelters	3
28	Gas concentration (NH_3, CO_2, H_2S)	NH_3 > 20 ppm, CO_2 > 3,000 ppm, H_2S > 0.5 ppm	1
		NH_3 = 10-20 ppm, CO_2 = 1,500-3,000 ppm, H_2S < 0.5 ppm	2
		NH_3 < 10 ppm, CO_2 < 1,500 ppm, H_2S < 0.5 ppm	3
29	Use of artificial lighting	no artificial lighting	1
		presence of artificial lighting	2
		presence of artificial lighting and adequate lighting (40 lux) during night-time to allow inspection of the animals	3
**Area C – Animal-based measures**			
N	***Item***	***Risk level***	
30	Avoidance distance test	animals cannot be approached as close as 100 cm	1

		animals can be approached as close as 100 to 50 cm but cannot be touched	2
		animals can be approached closer than 50 cm and can be touched	3
31	Performance of agonistic behaviour	frequent performance of agonistic behaviour (> 50% of the observed behaviours)	1
		moderate performance of agonistic behaviour (10-50% of the observed behaviours)	2
		low performance of agonistic behaviour (< 10% of the observed behaviours)	3
32	Body condition score (BCS)	> than 10% of the animals are very lean (BCS ≤ 2)	1
		2-10% of the animals are very lean (BCS ≤ 2)	2
		< than 2% of the animals are very lean (BCS ≤ 2)	3
33	Cleanliness of the animals	more than 40% of dirty animals	1
		10-40% of dirty animals	2
		less than 10% of dirty animals	3
34	Integument alterations	more than 20% of the animals with integument alterations	1
		10-20% of the animals with integument alterations	2
		less than 10% of the animals with integument alterations	3
35	Lameness	more than 6% of lame animals	1
		2-6% of lame animals	2
		less than 2% of lame animals	3
36	Severe respiratory disease (animals within the first 40 days since the arrival to the fattening unit)	more than 15% of the animals with severe respiratory disease	1
		5-15% of the animals with severe respiratory disease	2
		less than 5% of the animals with severe respiratory disease	3
37	Severe respiratory disease (animals from day 41 since the arrival to the fattening unit to slaughter)	more than 4% of the animals with severe respiratory disease	1
		2-4% of the animals with severe respiratory disease	2
		less than 2% of the animals with severe respiratory disease	3
38	Mild respiratory and/or enteric diseases (animals within the first 40 days since the arrival to the fattening unit)	more than 40% of the animals with mild respiratory and/or enteric diseases	1
		20-40% of the animals with mild respiratory and/or enteric diseases	2
		less than 20% of the animals with mild respiratory and/or enteric diseases	3
39	Mild respiratory and/or enteric diseases (animals from day 41 since the arrival to the fattening unit to slaughter)	more than 20% of the animals with mild respiratory and/or enteric diseases	1
		10-20% of the animals with mild respiratory and/or enteric diseases	2
		less than 10% of the animals with mild respiratory and/or enteric diseases	3
40	Annual mortality rate (animals died on-farm, or were euthanized or slaughtered due to an emergency)	annual mortality rate > 5%	1
		annual mortality rate between 2% and 5%	2
		annual mortality rate < 2%	3
41	Mutilations (e.g. disbudding, dehorning, tail docking)	presence of mutilations not allowed by national law (type of mutilation, procedure, age of the animal) (D. Lgs. N.146/2001)	1
		presence of mutilations allowed by national law (type	2

		of mutilation, procedure, age of the animal) (D. Lgs. N.146/2001)	
		absence of mutilations	3
42	Castration	castration is not allowed by national law (procedure, age of the animal) (D. Lgs. N.146/2001)	1
		castration is allowed by national law (procedure, age of the animal) (D. Lgs. N.146/2001)	2
		absence of castration	3
**SECTION - BIOSECURITY**			
N	***Item***	***Risk level***	
43	Rodent and insect control programmes	lack of measures	1
		minor measures	2
		good and effective measures supported by an accurate recording system	3
44	Measures to prevent the entrance of occasional visitors	lack of measures	1
		minor measures	2
		good and effective measures supported by an accurate recording system	3
45	Measures to control the entrance of regular visitors	no measures and no attention regarding visitors' boots and clothes	1
		upon entering the farm, all visitors must wear disposable boot covers or farm-specific boots and clothes	2
		upon entering the farm, all visitors are obliged to use a separate changing room to wear farm-specific and personal protective equipment (e.g. boots, clothes, gloves, boot/shoe covers)	3
46	Disinfection of vehicles (e.g. trucks, cars) upon entering the farm	lack of disinfection devices	1
		no specific disinfection devices and/or use of disinfection devices just in case of emergency	2
		all vehicles have to pass through specific and effective disinfection devices (e.g., clean transport bath, high pressure wash-down facility). They are always used.	3
47	Contact between external truck (e.g., feed truck) and cattle on farm	external trucks have direct/indirect contact with cattle (< 20 m distance)	1
		external trucks do not have direct/indirect contact with cattle (> 20 m distance)	2
48	Carcass storage and removing	lorries used to remove the carcasses have direct/indirect contact with cattle (< 20 m distance)	1
		carcasses are stored in a specific place at the edge of the farm, thus the lorries used to remove the carcasses do not enter the farm and do not have any direct/indirect contact with cattle (> 20 m distance)	2
49	Livestock truck (1)	livestock trucks that arrive on farm are not empty (animals from other farms are in the vehicle)	1
		livestock trucks that arrive on farm are always empty, clean and disinfected	2
50	Livestock truck (2)	animals are loaded close (< 20 m distance) to the areas where the other cattle are kept	1
		animals are loaded far from the areas (> 20 m distance) where the other cattle are kept	2

51	Quarantine	no quarantine for new cattle entering the barn	1
		minor quarantine measures (e.g., designated area not separated from the areas where the other cattle are kept, quarantine is too short, no biological tests)	2
		good and effective quarantine measures (e.g. designated area separated from the areas where the other cattle are kept, adequate duration of the quarantine, biological tests)	3
52	Analysis of drinking water	in case of drinking water, the quality of the water is not checked every year	1
		drinking water comes from the town supply system or from other sources and the quality of the water is checked at least once a year	2
**SECTION – EMERGENCY MANAGEMENT**			
N	***Item***	***Risk level***	
53	Noise exposure	cattle are exposed to a high level of noise	1
		cattle are exposed to a normal level of noise	2
54	Alarm system in case of mechanical ventilation disruption	absence of the alarm system	1
		presence of the alarm system	2
55	Fire alarm system	absence of the alarm system	1
		presence of the alarm system	2
56	Drinking water sources	only one drinking water source and no storage tank	1
		only one drinking water source but presence of a storage tank that guarantees a sufficient water supply in case of disruption of the water source	2
		presence of two or more drinking water sources	3

[1] Bertocchi, L. *et al*. Characterization of hazards, welfare promoters and animal-based measures for the welfare assessment of dairy cows: Elicitation of expert opinion. *Prev. Vet. Med*. **150**, 8-18 (2018).
